# Early macular microvascular attenuation in moderate myopia: projection artifact-removed and magnification-corrected optical coherence tomography angiography in an Iranian adult cohort

**DOI:** 10.1186/s12886-026-05057-4

**Published:** 2026-06-24

**Authors:** Afsaneh Naderi, Farshad Afshar, Kobra Nasrollahi, Heshmatollah Ghanbari, Ali Salehi, Alireza Ramezani Majd

**Affiliations:** Department of Ophthalmology, Feiz Eye Hospital, Isfahan University of Medical Sciences, Isfahan, Iran

**Keywords:** Myopia, OCT angiography, Vessel density, Projection artifact removal, Magnification correction, Deep capillary plexus

## Abstract

**Background:**

This study aimed to evaluate macular and peripapillary optical coherence tomography angiography (OCTA) parameters in mild and moderate myopic eyes compared with emmetropic controls, utilizing built-in projection artifact removal (PAR) and rigorous magnification correction.

**Methods:**

This prospective cross-sectional study included 218 eyes (90 emmetropic, 59 mild myopic, and 69 moderate myopic) of adults aged 20–52 years. Macular and optic disc OCTA scans were acquired using the RTVue-XR Avanti system with the built-in 3D-PAR algorithm enabled. Lateral magnification correction was performed post-hoc using the full Littmann–Bennett method ($\:t=p\times\:q$), with quadratic scaling applied to area measurements to ensure absolute mathematical precision. Eyes with axial length (AL) > 25.8 mm were excluded to minimize segmentation artifacts associated with posterior staphyloma and globe deformation.

**Results:**

Deep macular vessel density (VD) demonstrated a significant stepwise reduction across groups: emmetropia (45.92% ± 8.83) → mild myopia (41.44% ± 7.94) → moderate myopia (39.64% ± 10.74) (ANOVA *P* < 0.001). In contrast, superficial macular VD showed no statistically significant difference between mild myopia and emmetropia, with a significant decline observed only in moderate myopia (*P* < 0.05). The Foveal Avascular Zone (FAZ) area was significantly enlarged in moderate myopia (0.35 ± 0.12 mm², *P* < 0.001) but remained stable in mild myopia. In multivariable regression, spherical equivalent (SE) was the dominant independent predictor of deep VD reduction (Standardized β = -0.41, *P* < 0.001).

**Conclusions:**

Retinal microvascular attenuation appears to begin early in the deep capillary plexus (even in mild myopia), whereas superficial plexus and FAZ changes are features of more advanced severity. These findings suggest that DCP vessel density is a sensitive population-level indicator of early pathophysiological remodeling, although its current diagnostic utility for individual clinical monitoring is limited by measurement variability relative to the effect size.

**Supplementary Information:**

The online version contains supplementary material available at 10.1186/s12886-026-05057-4.

## Introduction

Myopia is rapidly becoming a global public health crisis, with prevalence rates projected to affect nearly 50% of the world’s population by 2050 [1]. While the sight-threatening complications of pathological high myopia—such as myopic maculopathy, choroidal neovascularization, and glaucoma—are well-documented [2, 3], the early subclinical microvascular alterations that occur between emmetropia to mild and moderate myopia remain incompletely understood.

Optical coherence tomography angiography (OCTA) has emerged as a powerful, non-invasive tool for visualizing the retinal microvasculature, allowing for the distinct assessment of the superficial capillary plexus (SCP) and the deep capillary plexus (DCP). Theoretical models suggest that the DCP, due to its complex angio-architecture and role in venous outflow, may be more vulnerable to the mechanical stretching and hemodynamic changes associated with axial elongation [4]. However, previous OCTA studies investigating the “dose-response” relationship between refractive error and vascular density have yielded inconsistent results [5, 6].

These discrepancies are largely attributable to two critical methodological challenges: projection artifacts and lateral magnification errors [7]. Projection artifacts from superficial vessels can erroneously inflate the signal in the deep plexus, masking true capillary loss [8]. Furthermore, the elongation of the globe in myopia introduces a magnification error that, if left uncorrected, leads to an underestimation of vessel density in eyes with longer axial lengths (AL) [9]. While prior studies have provided valuable insights [10, 11], conflicting findings regarding the specific onset of vascular attenuation in moderate myopia persist, partly due to varying methodologies in artifact removal and cohort definitions.

To address these limitations, this study aims to evaluate macular and peripapillary microvascular changes in an Iranian adult cohort, specifically comparing emmetropia, mild myopia, and moderate myopia. We employed a rigorous methodology combining the device’s built-in 3D Projection Artifact Removal (PAR) algorithm with post-hoc magnification correction using Bennett’s formula [12]. This approach allows us to determine whether microvascular attenuation in the deep plexus serves as an early pathophysiological indicator preceding the overt structural damage observed in advanced myopia.

## Methods

### Study design and participants

This prospective study with cross-sectional analysis was conducted at Feiz Eye Hospital, affiliated with Isfahan University of Medical Sciences, between 2022 and 2024. The study protocol adhered to the tenets of the Declaration of Helsinki and was approved by the Institutional Review Board (Ethics Code: IR.IUMS.REC.1401.123). Written informed consent was obtained from all participants prior to enrollment.

### Inclusion and exclusion criteria

Eligible participants were healthy adults aged ≥ 20 years. Inclusion criteria included a best-corrected visual acuity (BCVA) of 20/25 or better, intraocular pressure (IOP) < 21 mmHg, and astigmatism < 2.00 D.

Exclusion criteria were: (1) History of any ocular surgery (including refractive surgery) or trauma; (2) Evidence of retinal or optic nerve pathology (e.g., glaucoma, diabetic retinopathy, epiretinal membrane); (3) Systemic diseases known to affect the microvasculature (e.g., hypertension, diabetes mellitus); (4) Poor image quality defined as a Signal Strength Index (SSI) < 5 or significant motion artifacts.

Crucially, eyes with an axial length (AL) > 25.8 mm were excluded. This strict cutoff was applied not due to formula limitations, but to minimize the inclusion of eyes with posterior staphyloma or significant globe deformation. Such structural deformations can induce segmentation errors in the OCTA slab processing (Z-axis warping) that post-hoc magnification correction formulas cannot resolve, ensuring that observed changes are vascular rather than artifactual.

### Refractive grouping

Participants underwent a comprehensive ophthalmic examination, including cycloplegic refraction and axial length measurement (IOLMaster 500, Carl Zeiss Meditec). Eyes were classified into three groups based on spherical equivalent (SE):

- Emmetropia (*n* = 90): -0.50 D ≤ SE ≤ + 0.50 D.
- Mild Myopia (*n* = 59): -2.99 D ≤ SE < -0.50 D.
- Moderate Myopia (*n* = 69): -6.00 D < SE ≤ -3.00 D.

To control for inter-eye correlation, only one randomly selected eye per participant was included in the analysis.

### Image acquisition and processing

OCTA imaging was performed using the RTVue-XR Avanti system (Optovue Inc., Fremont, CA, USA). Macular (3 × 3 mm) and Optic Disc (4.5 × 4.5 mm) scans were acquired. The device’s built-in 3D Projection Artifact Removal (PAR) algorithm was enabled for all scans. This algorithm resolves projection artifacts from superficial vessels to improve the visualization and quantification of the deep capillary plexus (DCP).

Vessel density (VD) and foveal avascular zone (FAZ) area were calculated automatically using the device’s built-in AngioVue analytics software (version 2018.0.0.14). To ensure data reliability, manual quality control was performed on all B-scans. Only a small minority of scans (< 5%) required minor manual boundary corrections. To eliminate single-grader bias, a rigorous ‘Sequential Consensus Protocol’ was established: all manual adjustments were executed independently by an experienced grader and subsequently audited and finalized by a senior masked investigator until absolute agreement was reached. To quantitatively evaluate the reproducibility of these manual adjustments, a randomized subset of 30 corrected scans was blindly re-evaluated by both graders. The inter-grader Intraclass Correlation Coefficient (ICC) demonstrated excellent reliability, yielding 0.96 (95% CI: 0.92–0.98) for deep macular vessel density and 0.97 (95% CI: 0.94–0.99) for the superficial plexus (Supplementary Figure S1).

### Magnification correction

To account for ocular magnification variations induced by differences in axial length (AL), post-hoc lateral magnification correction was systematically applied to all quantitative OCTA metrics using the full Littmann–Bennett method [12] (t = p × q). The telecentric camera-specific constant (*p* = 3.382) was derived based on the standard schematic eye with a reference axial length (AL_reference) of 24.46 mm, rooted in the established optical calibration specifications for the Optovue RTVue-XR Avanti system [13].

Mathematically, this constant is defined as *p* = 1 / [0.01306 × (24.46–1.82)], where 1.82 mm denotes the distance from the corneal apex to the principal line. The participant-specific ocular repair factor (q) was calculated for each individual eye using the formula q = 0.01306 × (AL − 1.82).

The final comprehensive scaling factor (t) was applied linearly to correct distance-based parameters, whereas for area-based measurements—specifically the foveal avascular zone (FAZ) area—the correction factor was applied quadratically as t² = (p × q)².

A comprehensive sensitivity analysis comparing this primary absolute method against the simplified relative approach **(**q_relative = (AL − 1.82) / (24.46–1.82)**)** was conducted to verify consistency, as detailed in Supplementary Table S2 and Supplementary Figure S2.

### Statistical analysis

Statistical analyses were performed using SPSS software version 26 (IBM Corp, Armonk, NY). The normality of data distribution was assessed using the Shapiro-Wilk test. Comparisons of demographic and microvascular parameters across the three groups were performed using One-Way ANOVA, followed by Tukey’s post-hoc test for pairwise comparisons.

Pearson’s correlation analysis was used to evaluate the association between ocular biometry (AL, SE) and microvascular parameters. Multivariable linear regression was performed to identify independent predictors of vessel density. Crucially, the covariates included in the multivariable linear regression model—specifically Age, Signal Strength Index (SSI), and Optic Disc Area—were selected strictly a priori based on robust clinical, optical, and anatomical knowledge from established literature, rather than via post-hoc univariable screening or automated stepwise algorithms. Age was selected to control for age-related biological capillary dropout; SSI was included to account for technical variations in optical signal strength that artificially alter automated thresholding; and Optic Disc Area was included to control for individual macro-structural disc variations that confound capillary grid layouts. No post-hoc univariable P-value thresholds (e.g., *P* < 0.10) were employed for variable selection, ensuring that these clinically mandatory confounders remained preserved in the final model regardless of their univariable significance.

A P-value < 0.05 was considered statistically significant. For sectoral analyses involving multiple comparisons, the False Discovery Rate (FDR) was controlled using the Benjamini-Hochberg procedure.

## Results

### Demographic and clinical characteristics

A total of 218 eyes from 218 participants were included. The mean age was 34.10 ± 8.13 years in the emmetropic group, 32.59 ± 7.77 years in the mild myopic group, and 33.81 ± 8.02 years in the moderate myopic group (*P* = 0.16). Sex distribution was balanced (*P* = 0.22). As expected, SE and axial length (AL) showed significant differences across the three groups (*P* < 0.001) (Table 1).

**Table 1:** Demographic and clinical characteristics

Parameter	Emmetropia (*n* = 90)	Mild Myopia (*n* = 59)	Moderate Myopia (*n* = 69)	*P*-value (ANOVA)
Age (years)	34.10 ± 8.13	32.59 ± 7.77	33.81 ± 8.02	0.16
Sex (Male/Female)	44 / 46	30 / 29	35 / 34	0.22
Spherical Equivalent (D)	-0.39 ± 0.31	-2.00 ± 0.50	-4.50 ± 1.00	< 0.001*
Axial Length (mm)	23.72 ± 0.74	24.20 ± 0.60	24.44 ± 0.81	< 0.001*
Signal Strength Index (SSI)	7.15 ± 0.38	7.02 ± 0.41	7.05 ± 0.39	0.40

*Indicates statistical significance (*P* < 0.05)

Values are presented as Mean  ±  Standard Deviation (SD)

### Macular microvascular analysis

Table 2 summarizes the vessel density (VD) findings after magnification correction.

**Table 2:** Macular and peripapillary vessel density (Magnification-Corrected)

Parameter	Emmetropia (*n* = 90)	Mild Myopia (*n* = 59)	Moderate Myopia (*n* = 69)	*P*-value (ANOVA)	Post-hoc (Mild vs. Mod)
Deep Macular VD	45.92 ± 8.83	41.44 ± 7.94	39.64 ± 10.74	< 0.001*	< 0.001
Superficial Macular VD	47.52 ± 4.56	44.90 ± 5.20	45.88 ± 4.92	0.025*	0.38
Inside-Disc VD	48.20 ± 7.02	45.15 ± 7.53	46.10 ± 7.12	0.08	—
Nasal Peripapillary VD	48.45 ± 5.48	49.56 ± 3.78	46.79 ± 5.55	0.09	—

Note: Post-hoc pairwise comparisons were not performed for peripapillary parameters as the omnibus ANOVA was not statistically significant

Values are Mean  ±  SD (%). Comparisons performed using One-Way ANOVA with Tukey’s post-hoc test

#### Deep Capillary Plexus (DCP)

**Fig. 1:**
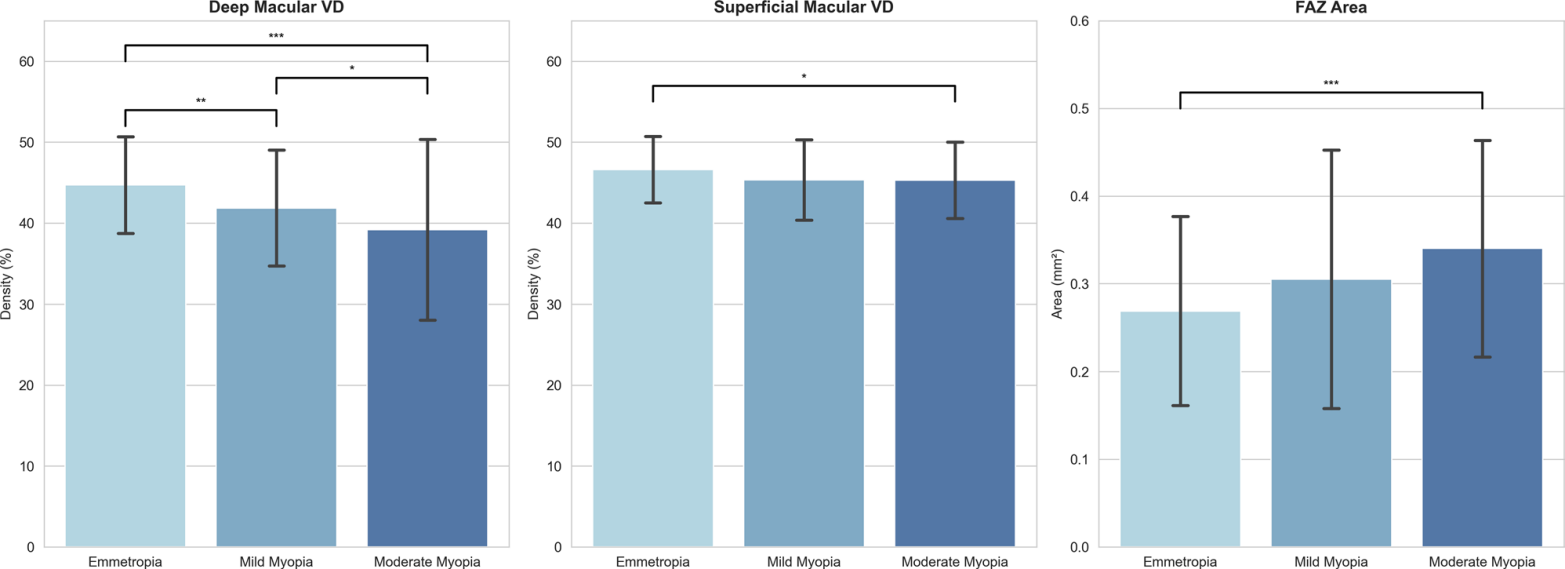
Comparison of microvascular parameters across refractive groups. Bar charts displaying the mean values with standard deviation error bars for Emmetropia (*n* = 90), Mild Myopia (*n* = 59), and Moderate Myopia (*n* = 69). (**a**) Deep Macular VD: Shows a significant stepwise reduction from Emmetropia to Mild Myopia (**P < 0.01) and from Mild to Moderate Myopia (**P < 0.01). (**b**) Superficial Macular VD: Shows relative stability in the Mild group (ns), with a significant reduction observed only in the Moderate group compared to Emmetropia (*P < 0.05). (**c**) FAZ Area: Shows significant enlargement only in the Moderate Myopia group (***P < 0.001)

A significant stepwise reduction was observed in whole-image deep macular VD across the refractive groups (ANOVA *P* < 0.001) (Fig. 1). Post-hoc Tukey analysis confirmed that deep VD in mild myopia (41.44 ± 7.94%) was significantly lower than in emmetropia (45.92 ± 8.83%, *P* = 0.004) and significantly higher than in moderate myopia(39.64 ± 10.74%, *P* < 0.001).Detailed sectoral analysis of the deep capillary plexus is presented in Table 3.

**Table 3:** Sectoral deep macular vessel density (FDR-Adjusted)

Deep Plexus Sector	Emmetropia (*n* = 90)	Moderate Myopia (*n* = 69)	Raw *P*-value	FDR Adjusted q-value
Fovea	22.1 ± 5.2	20.8 ± 5.5	0.042*	0.048*
Parafovea Temporal	48.3 ± 7.1	42.1 ± 8.3	< 0.001*	< 0.001*
Parafovea Superior	47.8 ± 6.9	41.5 ± 8.1	< 0.001*	< 0.001*
Parafovea Nasal	46.5 ± 7.5	40.9 ± 8.5	< 0.001*	< 0.001*
Parafovea Inferior	47.2 ± 7.3	41.2 ± 8.2	< 0.001*	< 0.001*

FDR: False Discovery Rate (Benjamini-Hochberg procedure)

Values are Mean  ±  SD (%). Comparison focuses on the primary transition from Emmetropia to Moderate Myopia

#### Superficial Capillary Plexus (SCP)

The overall ANOVA showed a significant difference (*P* = 0.025). However, unlike the deep plexus, there was no statistically significant difference between mild myopia (44.90 ± 5.20%) and emmetropia (47.52 ± 4.56%, *P* > 0.05). A significant decline was observed only in the moderate myopia group compared to emmetropia (*P* < 0.05).

#### **Foveal Avascular Zone (FAZ)**

The FAZ area remained stable in the early stages (emmetropia: 0.28 ± 0.11 mm² vs. Mild: 0.29 ± 0.15 mm², P > 0.05). Significant enlargement was observed only in moderate myopia (0.35 ± 0.12 mm², P < 0.001).

### Peripapillary microvasculature

In the peripapillary region, the Inside-Disc VD (*P* = 0.08) and Nasal Peripapillary VD (*P* = 0.09) did not differ significantly across groups in the omnibus ANOVA. Consequently, no post-hoc pairwise comparisons were performed for these parameters.Structural parameters, including RNFL and GCC thickness, are summarized in Table 4.

**Table 4:** Structural parameters

Parameter	Emmetropia (*n* = 90)	Mild Myopia (*n* = 59)	Moderate Myopia (*n* = 69)	*P*-value (ANOVA)
Nasal RNFL (µm)	99.64 ± 20.09	97.69 ± 14.85	90.38 ± 20.15	0.002*
GCC Average (µm)	97.73 ± 5.77	96.70 ± 5.75	95.12 ± 6.10	0.008*
FAZ Area (mm²)	0.28 ± 0.11	0.29 ± 0.15	0.35 ± 0.12	< 0.001*

*Indicates statistical significance (*P* < 0.05)

Values are Mean  ±  SD. Comparisons performed using One-Way ANOVA

### Correlation and multivariable regression

**Fig. 2:**
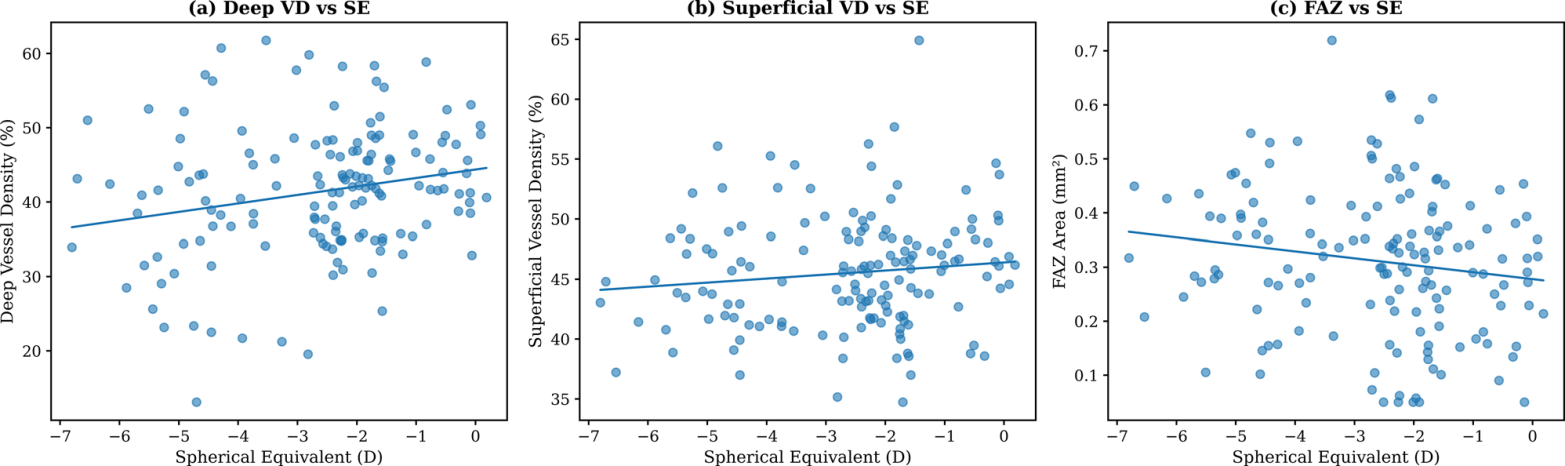
Correlations between Spherical Equivalent (SE) and microvascular parameters. Scatter plots demonstrating the relationship between refractive error and microvascular metrics across the entire study cohort (*n* = 218).Values in the plots represent univariable Pearson correlation coefficients (r). For adjusted independent effects, refer to the standardized β values in Table 5. (**a**) Deep Macular Vessel Density (VD) shows a significant positive correlation with SE (Pearson’s *r* = 0.489, *P* < 0.001), indicating that lower SE (more myopic) correlates with reduced density. (**b**) Superficial Macular VD shows a weaker association. (**c**) FAZ Area shows a negative correlation with SE (*r* = -0.312, *P* = 0.002), indicating enlargement as myopia worsens. The solid lines represent the linear regression fit

In univariable correlation analysis involving the entire cohort, deep macular VD demonstrated a significant positive correlation with SE (Pearson’s *r* = 0.489, *P* < 0.001; Fig. 2a), confirming that a lower spherical equivalent (more myopic refractive error) tracks directly with reduced capillary density. Concurrently, axial length (AL) exhibited a significant negative correlation with deep macular VD (Pearson’s *r* = − 0.294, *P* = 0.003), mathematically demonstrating that longer axial dimensions are associated with lower vessel density. As expected, SE and AL were strongly inversely correlated (Pearson’s *r* ≈ − 0.75, *P* < 0.001), and Fig. 2 illustrates these consistent biometrical relationships.

To distinguish between simple associations and independent effects, we reported both univariable correlation coefficients (r) and adjusted standardized coefficients from the multivariable model.

To adjust for potential confounders, a multivariable linear regression analysis was performed (Table 5). After adjusting for Age, SSI, and Disc Area, SE remained the dominant independent predictor of Deep Macular VD (Standardized β= -0.41, *P* < 0.001), while the independent effect of AL was attenuated (*P* = 0.21) due to the high collinearity with SE.Notably, since SE was entered into the model as absolute magnitudes, this negative β coefficient reflects the pathophysiological relationship where higher degrees of myopic refractive error are independently associated with lower vessel density.

**Table 5:** Multivariable linear regression analysis for deep macular VD

Independent Predictor	Unstandardized B (SE)	Standardized β	95% CI for B	*P*-value	VIF
Spherical Equivalent (SE)	-1.85 (0.38)	-0.41	-2.61 to -1.09	< 0.001*	1.82
Axial Length (AL)	-0.52 (0.41)	-0.11	-1.41 to 0.37	0.21	1.71
Age	-0.08 (0.05)	-0.09	-0.18 to 0.02	0.11	1.05
Signal Strength (SSI)	2.15 (0.95)	0.14	0.28 to 4.02	0.024*	1.03

Note: SE values were entered as absolute magnitudes in the regression model; thus, a negative beta coefficient indicates that greater myopic severity is independently associated with decreased vessel density

Model Summary: Adjusted R² = 0.29, *P* < 0.001. VIF: Variance Inflation Factor

*Indicates statistical significance (*P* < 0.05)

Dependent Variable: Deep Macular Vessel Density (Whole Image). Adjusted for Age, SSI, and Disc Area

## Discussion

This study demonstrates a distinct, layer-specific vulnerability of the retinal microvasculature during the early stages of myopic severity.The primary finding is a stepwise reduction in deep macular vessel density that initiates in mild myopia, preceding changes in the superficial plexus and foveal avascular zone. This suggests that the deep capillary plexus may be the first vascular bed to reflect the mechanical and hemodynamic stress of axial elongation.

### Deep capillary plexus: The early responder

The most significant finding of the current study is the early and progressive loss of deep macular vessel density. We observed that deep VD was significantly reduced even in the mild myopia group compared to emmetropic controls. This vulnerability may be attributed to the unique angio-architecture of the DCP; it consists of finer capillaries with thinner walls and lower perfusion pressure compared to the superficial plexus, rendering it more susceptible to the mechanical stretching and hemodynamic changes associated with early axial elongation [4, 10].

It is important to note that while the observed 6.3% reduction in deep VD is statistically significant at the group level, it remains slightly below the previously reported minimum detectable change (MDC) of approximately 7.45% for this device. Therefore, these changes should be interpreted as population-level pathophysiological trends rather than a definitive diagnostic tool for individual clinical monitoring at this stage.

Our observation of selective deep plexus vulnerability aligns with Al-Sheikh et al. [10], who similarly reported significant DCP attenuation as a primary feature of myopic remodeling. However, our findings contrast with other reports that identified early changes predominantly in the superficial plexus or found no significant layer-specific differences [5, 6]. These discrepancies likely stem from the “projection artifact” phenomenon, where superficial vessels cast shadows on the deep layers, artificially inflating the deep vessel density signal and masking early dropouts. By utilizing the 3D-PAR algorithm in this study, we effectively eliminated these confounding signals, unmasking the true vulnerability of the deep capillary plexus in mild myopia which might have been overlooked in studies lacking rigorous artifact removal.

This contrast between the DCP and SCP involvement further supports the ‘layer-specific’ susceptibility theory in early myopic remodeling [4, 10]. In our cohort, superficial macular VD exhibited a minor, non-linear numerical fluctuation between mild (44.90% ± 5.20%) and moderate myopia (45.88% ± 4.92%). This small numerical reversal was statistically negligible (*P* = 0.38) and did not represent a true clinical or pathophysiological trend. We hypothesize that this observation is driven by two concurrent factors. First, it reflects standard measurement noise and high individual variance within the cohorts; this 1% numerical variance falls well within the device’s known test-retest coefficient of variation (2.6%), as shown in our quality control data (Supplementary Table S1) [5, 11].

Second, it underscores the relative biomechanical stability of the superficial capillary plexus (SCP) during the initial stages of axial elongation. The SCP, being embedded within the structurally robust matrix of the peripapillary retinal nerve fiber layer (RNFL) and ganglion cell layer (GCL), benefits from greater physical scaffolding and autoregulatory protection compared to the thinner, highly vulnerable deep capillary plexus (DCP) [4, 8].

The application of rigorous 3D-PAR was essential in unmasking this layer-specific divergence [8], confirming that early myopic microvascular attenuation is primarily restricted to the deep plexus, while the superficial plexus remains structurally compensated or directionally stable during early myopic progression.

In the peripapillary region, our analysis revealed that both Inside-Disc VD (*P* = 0.08) and Nasal Peripapillary VD (*P* = 0.09) did not achieve statistical significance across the refractive tiers. Mechanistically, this relative resistance of the peripapillary microvasculature can be attributed to the unique anatomical architecture of the radial peripapillary capillaries (RPCs). The RPCs are deeply embedded within a densely packed axonal scaffold of the thick peripapillary retinal nerve fiber layer (RNFL) and its supporting astrocytic sheath [11]. This robust structural matrix acts as a biomechanical cushion, effectively shielding the peripapillary capillary beds from the initial tensile and mechanical strains induced by mild-to-moderate axial elongation compared to the more delicate macular networks [4, 8]. Furthermore, by adjusting for Optic Disc Area a priori in our multivariable models, we effectively eliminated macro-anatomical confounding, thereby unmasking the true physiological stability of the peripapillary network during these early refractive transitions.

### Methodological rigor

Our study differentiates itself by strictly excluding eyes with AL > 25.8 mm. This exclusion was not due to the limitations of magnification formulas, but rather to avoid the confounding effects of posterior staphyloma and globe deformation common in longer eyes. Such deformations can cause physical warping of the retinal layers, leading to segmentation errors that cannot be corrected mathematically. By controlling for this, we ensure that the observed vascular attenuation is a true physiological change.

### Limitations

This study has certain limitations. First, its cross-sectional design represents differences across refractive groups rather than temporal progression; therefore, no causal inferences or implications of ‘progression’ should be made. Second, although manual segmentation corrections were required in less than 5% of the cohort, our reliance on automated software boundaries means minor subclinical segmentation noise could theoretically persist, though this risk was strictly minimized using a dual-grader consensus protocol with verified high inter-grader reliability.

Finally, although we used rigorous artifact removal and magnification correction, the findings should be validated in longitudinal cohorts.

## Conclusion

In conclusion, cross-sectional retinal microvascular density exhibits a severity-dependent, layer-specific variation across refractive tiers. This is characterized by lower deep capillary plexus (DCP) density in mild myopia, while superficial plexus and FAZ alterations are apparent only at the moderate myopic tier. These findings highlight the deep plexus as a sensitive population-level indicator of early microvascular variations across severity levels, though future longitudinal cohort studies remain required to track true temporal progression and causal mechanisms.

## Supplementary Information

Below is the link to the electronic supplementary material.

Supplementary Material 1

## Data Availability

The datasets used and/or analyzed during the current study are available from the corresponding author on reasonable request.

## Declarations

### Use of artificial intelligence (AI)

Large language models (LLMs) were used solely for grammatical editing and language refinement during the preparation of this manuscript. The authors reviewed and edited the output and take full responsibility for the content of the publication.

## Abbreviations

AL: Axial length
DCP: Deep capillary plexus
FAZ: Foveal avascular zone
GCC: Ganglion cell complex
OCTA: Optical coherence tomography angiography
PAR: Projection artifact removal
RNFL: Retinal nerve fiber layer
SCP: Superficial capillary plexus
SE: Spherical equivalent
SSI: Signal strength index
VD: Vessel density
