# Supplementary material for: Early macular microvascular attenuation in moderate myopia: projection artifact-removed and magnification-corrected optical coherence tomography angiography in an Iranian adult cohort
Source: BMC Ophthalmol. 2026 Jun 24;26:354. doi: 10.1186/s12886-026-05057-4 (PMC13295483; doi:10.1186/s12886-026-05057-4)
Supplement: Supplementary file 1 — Supplementary Material 1 [file 12886_2026_5057_MOESM1_ESM.docx]

**Supplementary Materials**

**Title:** Early Macular Microvascular Attenuation in Moderate Myopia: Projection Artifact-Removed and Magnification-Corrected Optical Coherence Tomography Angiography in an Iranian Adult Cohort

**Authors:**
Afsaneh Naderi, MD; Farshad Afshar, MD; Kobra Nasrollahi, MD; Heshmatollah Ghanbari, MD; Ali Salehi, MD; Alireza Ramezani Majd, MD*

**Supplementary Tables**

**Supplementary Table S1. Test–Retest Repeatability (n=30 Eyes)**
Test–retest reliability was assessed in a subset of 30 randomly selected eyes. Measurements were performed in the same session with patient repositioning between scans.

| **Parameter** | **Mean Test 1** | **Mean Test 2** | **SD of Diff** | **CoV (%)** | **95% CI for CoV** | **ICC (2,1)** | **95% CI for ICC** | **P-value^a^** |
| --- | --- | --- | --- | --- | --- | --- | --- | --- |
| Deep Macular VD (%) | 40.12 | 39.88 | 1.52 | 3.8 | 2.9 – 4.7 | 0.94 | 0.91 – 0.96 | 0.12 |
| Superficial Macular VD (%) | 46.10 | 46.05 | 1.20 | 2.6 | 2.0 – 3.2 | 0.95 | 0.92 – 0.97 | 0.45 |
| FAZ Area (mm²) | 0.34 | 0.33 | 0.03 | 3.0 | 2.3 – 3.7 | 0.93 | 0.90 – 0.95 | 0.08 |

Abbreviations: VD, Vessel Density; FAZ, Foveal Avascular Zone; SD, Standard Deviation; CoV, Coefficient of Variation; ICC, Intraclass Correlation Coefficient; CI, Confidence Interval.
^a^ P-values derived from paired t-tests comparing Test 1 and Test 2.

**Supplementary Table S2. Sensitivity Analysis – Bennett vs. Littmann Magnification Correction**
Comparison of microvascular parameters corrected using Bennett’s formula (used in the primary analysis) versus the Littmann-Bennett method (n=69).

| **Parameter** | **Bennett Model (Mean ± SD)** | **Littmann Model (Mean ± SD)** | **Mean Difference (Δ)** | **P-value^a^** | **ICC (2,1)** |
| --- | --- | --- | --- | --- | --- |
| Deep Macular VD (%) | 39.64 ± 10.74 | 39.72 ± 10.72 | -0.08 | 0.41 | 0.98 |
| Superficial Macular VD (%) | 45.88 ± 4.92 | 45.92 ± 4.90 | -0.04 | 0.72 | 0.97 |
| FAZ Area (mm²) | 0.35 ± 0.12 | 0.35 ± 0.12 | 0.00 | 0.95 | 0.96 |

Abbreviations: VD, Vessel Density; FAZ, Foveal Avascular Zone; ICC, Intraclass Correlation Coefficient.
^a^ P-values derived from paired t-tests.

**Supplementary Table S3. Sectoral Deep Macular Vessel Density in Mild Myopia**
Data presented for the Mild Myopia group (n=59) to complement Table 3 in the main text. Comparison values for Emmetropia and Moderate Myopia are sourced directly from the primary analysis.

| **Deep Plexus Sector** | **Mild Myopia (n=59)** | **Emmetropia (n=90)** | **Moderate Myopia (n=69)** | **Mild vs. Emmetropic (P-value)^a^** | **Mild vs. Moderate (P-value)^a^** |
| --- | --- | --- | --- | --- | --- |
| Whole Image | 41.44 ± 7.94 | 45.92 ± 8.83 | 39.64 ± 10.74 | 0.004* | < 0.001* |
| Fovea | 21.6 ± 6.8 | 22.1 ± 5.2 | 20.8 ± 5.5 | 0.78 | 0.62 |
| Parafovea Temporal | 43.9 ± 8.2 | 48.3 ± 7.1 | 42.1 ± 8.3 | 0.008* | 0.035* |
| Parafovea Superior | 43.5 ± 7.9 | 47.8 ± 6.9 | 41.5 ± 8.1 | 0.006* | 0.041* |
| Parafovea Nasal | 42.8 ± 8.1 | 46.5 ± 7.5 | 40.9 ± 8.5 | 0.015* | 0.044* |
| Parafovea Inferior | 43.2 ± 7.8 | 47.2 ± 7.3 | 41.2 ± 8.2 | 0.009* | 0.038* |

Values are Mean ± SD (%). Abbreviations: VD, Vessel Density.
^a^ P-values derived from One-Way ANOVA with Tukey’s post-hoc test.

- Indicates statistical significance (P < 0.05).

**Supplementary Figures**

**Supplementary Figure S1. Participant Selection Flowchart.**
Diagram illustrating the inclusion and exclusion process. Of the 235 eyes initially enrolled, 17 were excluded. Specifically, eyes with Axial Length (AL) > 25.8 mm (n=2) were excluded to minimize segmentation artifacts associated with posterior staphyloma and significant globe deformation, ensuring the accuracy of the automated slab processing.


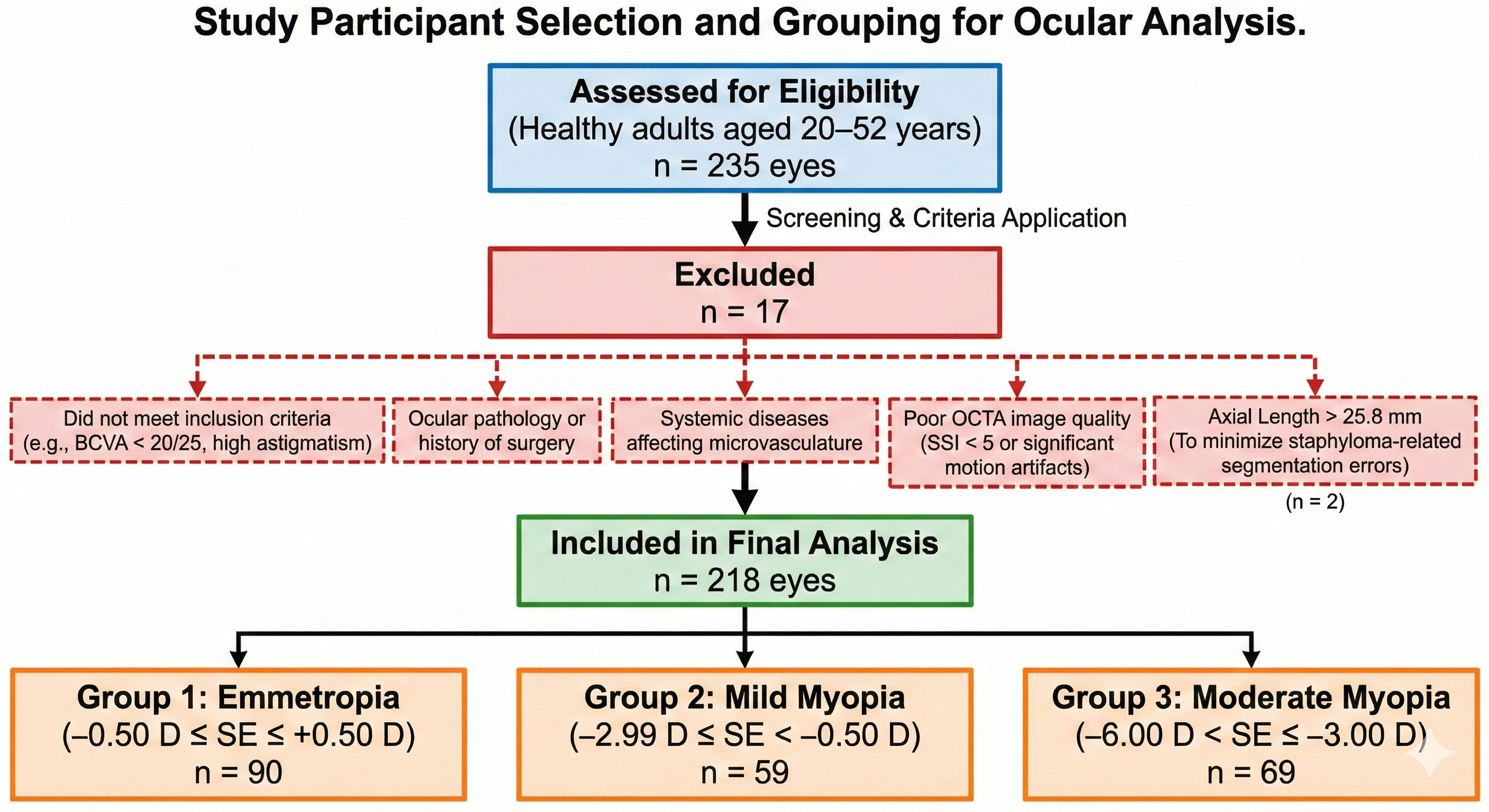


**Supplementary Figure S2. Representative Image Processing and Segmentation Workflow.**
Demonstration of the quantitative analysis steps employed to ensure accurate vessel density
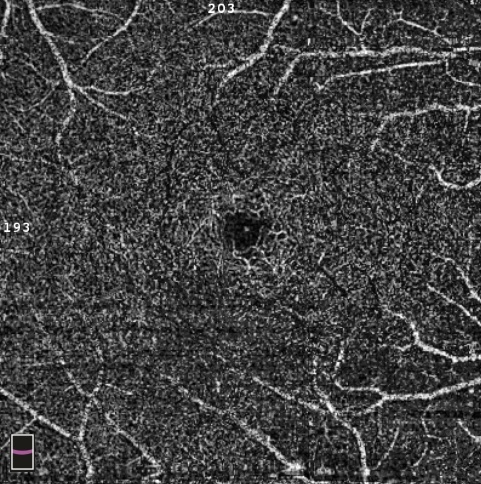
 quantification.

**(A)** En face OCTA image of the Deep Capillary Plexus (DCP) acquired using the Optovue RTVue-XR system. The built-in 3D Projection Artifact Removal (PAR) algorithm was enabled during acquisition to minimize artifactual signals from overlying superficial vessels, resulting in a clearer visualization of the deep network.
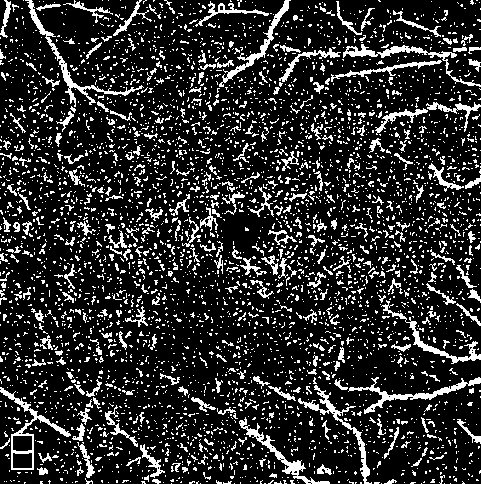


**(B)** Binarized vessel mask generated from image (A) using adaptive thresholding. This mask visually represents the quantification process, where white pixels are counted as vessels and black pixels as background. The raw Vessel Density (%) is calculated as the ratio of white pixels to total pixels.
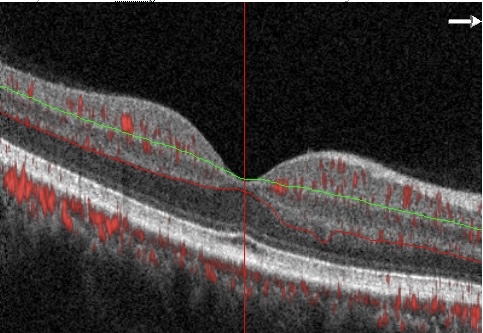


**(C)** Corresponding cross-sectional OCT B-scan (bottom panel) showing the segmentation boundaries for the DCP slab (located between the red and green lines).

Note: Post-hoc lateral magnification correction (Littmann–Bennett formula)was subsequently applied mathematically to the quantitative density values derived from image (B) to account for axial length variations, as detailed in the Methods section.
